# Supplementary material for: Causal Relationship between Aging and Anorexia Nervosa: A White-Matter-Microstructure-Mediated Mendelian Randomization Analysis
Source: Biomedicines. 2024 Aug 16;12(8):1874. doi: 10.3390/biomedicines12081874 (PMC11351342; doi:10.3390/biomedicines12081874)
Supplement: Supplementary file 1 [file biomedicines-12-01874-s001.zip › Supplementary Figure/Figure S4/Singe SNP plot of MR analysis for TL to ubm-b-1991.pdf]

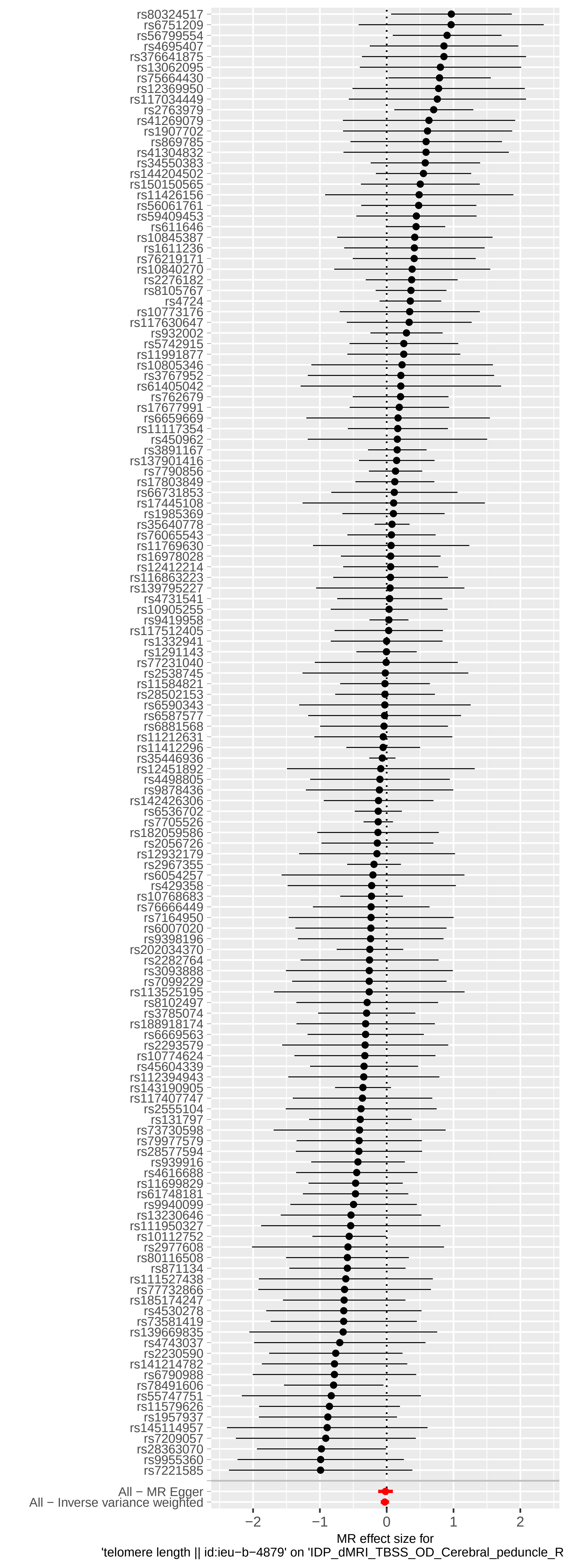
rs80324517
rs6751209
rs56799554
rs4695407
rs376641875
rs13062095
rs75664430
rs12369950
rs117034449
rs2763979
rs41269079
rs1907702
rs869785
rs41304832
rs34550383
rs144204502
rs150150565
rs11426156
rs56061761
rs59409453
rs611646
rs10845387
rs1611236
rs76219171
rs10840270
rs2276182
rs8105767
rs4724
rs10773176
rs117630647
rs932002
rs5742915
rs11991877
rs10805346
rs3767952
rs61405042
rs762679
rs17677991
rs6659669
rs11117354
rs450962
rs3891167
rs137901416
rs7790856
rs17803849
rs66731853
rs17445108
rs1985369
rs35640778
rs76065543
rs11769630
rs16978028
rs12412214
rs116863223
rs139795227
rs4731541
rs10905255
rs9419958
rs117512405
rs1332941
rs1291143
rs77231040
rs2538745
rs11584821
rs28502153
rs6590343
rs6587577
rs6881568
rs11212631
rs11412296
rs35446936
rs12451892
rs4498805
rs9878436
rs142426306
rs6536702
rs7705526
rs182059586
rs2056726
rs12932179
rs2967355
rs6054257
rs429358
rs10768683
rs76666449
rs7164950
rs6007020
rs9398196
rs202034370
rs2282764
rs3093888
rs7099229
rs113525195
rs8102497
rs3785074
rs188918174
rs6669563
rs2293579
rs10774624
rs45604339
rs112394943
rs143190905
rs117407747
rs2555104
rs131797
rs73730598
rs79977579
rs28577594
rs939916
rs4616688
rs11699829
rs61748181
rs9940099
rs13230646
rs111950327
rs10112752
rs2977608
rs80116508
rs871134
rs111527438
rs77732866
rs185174247
rs4530278
rs73581419
rs139669835
rs4743037
rs2230590
rs141214782
rs6790988
rs78491606
rs55747751
rs11579626
rs1957937
rs145114957
rs7209057
rs28363070
rs9955360
rs7221585
All − MR Egger
All − Inverse variance weighted
−2
−1
0
1
2
MR effect size for
'telomere length || id:ieu−b−4879' on 'IDP_dMRI_TBSS_OD_Cerebral_peduncle_R
